# Solid Cell Nests Within a Parathyroid Gland—Report of an Exceptional Case

**DOI:** 10.1007/s12022-018-9539-2

**Published:** 2018-07-12

**Authors:** C. Christofer Juhlin, Inga-Lena Nilsson, Anders Höög

**Affiliations:** 1 Department of Pathology and Cytology, Karolinska University Hospital, Stockholm, Sweden; 2 Department of Oncology-Pathology, Cancer Centre Karolinska (CCK) R8:04, Karolinska Institutet, 17176 Stockholm, Sweden; 3 Department of Molecular Medicine and Surgery, Karolinska Institutet, Stockholm, Sweden; 4 Department of Breast, Endocrine Tumors and Sarcoma, Karolinska University Hospital, Stockholm, Sweden

**Keywords:** Solid cell nest, Ultimobranchial body, Parathyroid

## Abstract

The ultimobranchial body (UBB) denotes the cellular mass originating from the fourth branchial pouch, which migrates from the neural crest and infolds within the middle and upper poles of the thyroid lobes, thereby establishing the presence of calcitonin-secreting parafollicular C cells. In various numbers, UBB remnants (entitled “solid cell nests”, or SCNs) are found in thyroid glands examined histologically. However, despite the close embryological relation between the UBB and the superior parathyroid glands, intraparathyroidal SCNs have to our knowledge not been previously reported. Here, we describe a patient presenting with a papillary thyroid carcinoma with central and lateral lymph node metastases. Upon postoperative analysis, an unintentionally removed parathyroid gland was observed adjacent to the superior aspect of the right thyroid lobe. Within a 0.6 × 0.5-mm area of the parathyroid gland, solid nests composed of epithelial cells with oval and slightly elongated nuclei were seen. The cells were positive for p40, p63, and GATA3, but negative for PTH. The final diagnosis was a SCN entrapped within the parathyroid gland. Empirically, we have not previously observed SCNs within the parathyroid glands. To our knowledge, our finding thus constitutes a very unusual histological manifestation, and could indicate an underlying aberrancy during embryogenesis given the close anatomical relationship between the UBB and the superior parathyroid glands.

## Introduction

The ultimobranchial body (UBB) appears as an out-pouching of the fourth pharyngeal pouch during the fifth week of embryonic development, by some described as a transient fifth pouch. At this stage, rudiments of the superior parathyroid glands are also visible in the fourth pouch [1]. The UBB and superior parathyroids detach from the pharyngeal wall, begin a medio-caudal migration between developmental weeks 5–7 and attach to the dorsal surface of the thyroid. The UBB cells are then dispersed across the superior and middle aspects of the thyroid lobes, and the cells later differentiate into parafollicular C cells, whereas the superior parathyroid glands implant as a functional unit [1].

UBB remnants (denoted solid cell nests, or SCNs) are recurrently found in routine histological assessments of the thyroid gland, and the frequency of this phenomenon in adult thyroid tissue ranges from 3 to 60%, with the latter numbers derived from serial sectioning studies in which the entire thyroid is submitted for histology [2–7]. Traditionally, the SCNs are divided into “main cells” or “C cells,” in which the main cells are the most predominant, with a squamoid histological appearance and exhibiting widespread and diffuse p63 positivity joined by TTF1 and calcitonin negativity, whereas the C cell type is positive for calcitonin and TTF1, but p63 negative [8–10]. Of late, GATA3 expression has also been reported in the majority of SCNs examined [11]. Due to their assumed pluripotent stem cell abilities, SCNs are hypothesized to be the origin of uncommon thyroid malignancies, such as “carcinoma showing thymus-like differentiation” (CASTLE) and primary mucoepidermoid carcinoma [9, 12–15]. However, apart from these observations, an eventual physiological role of SCNs in the adult thyroid gland remains partly obscure.

SCNs have not been reported in other organs apart from the thyroid except for rare manifestations with focal findings in the heart and in a single case of struma ovarii, in the former denoted as an “ultimobranchial heterotopia” [16, 17]. As SCNs are important to recognize in the clinical setting as the differential diagnoses include C cell hyperplasia, medullary thyroid carcinoma, and squamous cell carcinoma (primary or metastatic), the finding of these cell structures outside of the thyroid would be of both clinical and anatomical interest.

## Case Presentation

The patient is a 42-year-old female of Swedish ethnicity and no previous medical history. In 2018, she developed an enlarged lymph node in the right lateral aspect of the neck, and a subsequent fine-needle biopsy was consistent with metastatic papillary thyroid carcinoma (PTC). Thyroid ultrasonography visualized a focal lesion, 8 mm in diameter, located in the cranial part of the right lobe, but a fine needle aspiration biopsy only gave a bloody exchange, and no cytological diagnosis of the primary tumor was obtained. The patient underwent total thyroidectomy plus central and lateral lymph node dissection, and the histopathological examination revealed an 11-mm conventional PTC in the superior aspect of the right thyroid lobe. The tumor did not exhibit extrathyroidal extension and was radically removed. Moreover, lymph node metastases to the cervical (8/10 positive nodes) and lateral (9/24 positive nodes) compartments respectively were observed. Close to the primary tumor, a 3-mm parathyroid gland adjacent to the thyroid capsule was visualized, with focal findings that caught our interest for a more detailed analysis. Immunohistochemistry was performed using standardized protocols used in clinical routine.

## Histopathology

**Fig. 1:**
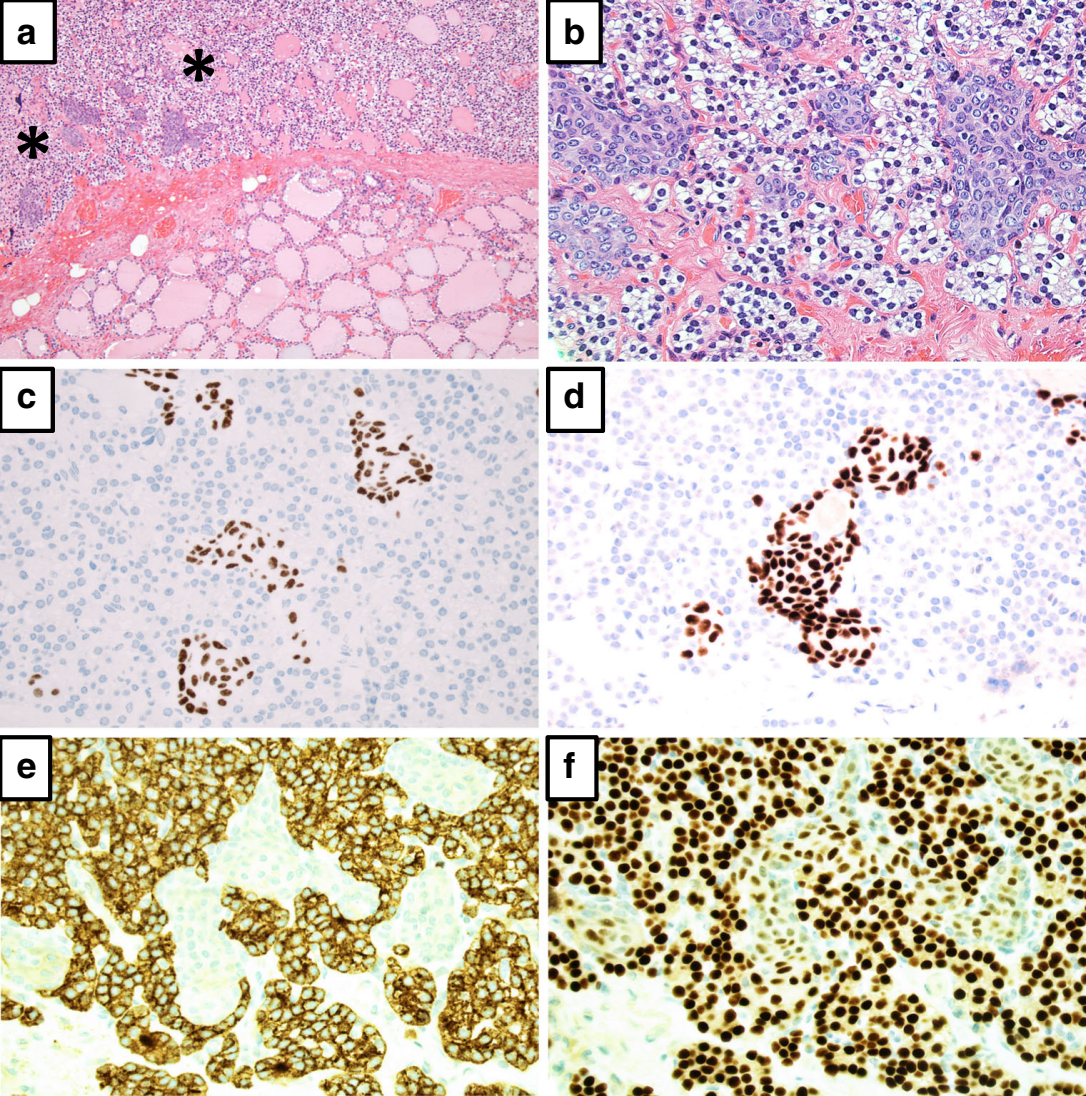
Histological and immunohistochemical profile of the parathyroid SCNs. **a** Hematoxylin-eosin stain at × 100 magnification depicting the parathyroid (upper part) and thyroid glands (lower part) respectively, with the intra-parathyroidal SCNs marked with asterisks. **b** Hematoxylin-eosin stain at × 400 magnification showing the cytomorphology of the SCN cells. **c**, **d** Immunohistochemistry visualizing distinct p40 and p63 immunoreactivity respectively. **e** PTH immunohistochemistry demonstrating absent staining in SCNs, but diffusely and strong cytoplasmic immunoreactivity in the surrounding parathyroid cells. **f** Immunohistochemistry with a GATA3 antibody reveals expected strong nuclear staining for parathyroid cells and weak positive staining in the nuclei of the SCN cells

Within a 0.6 × 0.5-mm large area of the parathyroid gland, multifocal groups of epithelial cells with oval and slightly elongated nuclei were easily detected using light microscopy and routine staining (Fig. 1a, b). The appearance was strikingly similar to the SCNs recurrently found in histological assessment of the thyroid gland. The nests were devoid of lymphocytic infiltrates and Hassall’s corpuscles, thereby excluding thymus tissue. No keratinization or intracellular bridges were seen, which would argue against a rare manifestation of squamous metaplasia. No mitoses were observed, contradicting a metastatic epithelial malignancy. Immunohistochemical analyses showed that the cells were positive for p40, p63, and GATA3, whereas no immunoreactivity was seen for parathyroid hormone (PTH). Immunostainings for TTF1, thyroglobulin, calcitonin, galectin-3, Bcl-2, and Ki-67 were uninformative due to cutting-away of the area of interest. The immunohistochemical findings are exemplified in Fig. 1c–f. The surrounding parathyroid tissue was strongly PTH positive, thereby excluding the possibility that the nests were in fact positioned within the thyroid. The findings thus most likely represent SCNs within a parathyroid gland.

The parathyroid tissue itself was surprisingly devoid of intraparenchymal adipose tissue, and focal areas of the gland exhibited an acinar growth pattern rather than the nuclear palisading recurrently seen in normal parathyroids. The final pathology report therefore highlights the difficulties in assessing whether this parathyroid gland was pathological or not. However, the normocalcemic state of the patient prior to surgery and the limited glandular size both argue against the development of an adenoma.

The surrounding normal thyroid tissue did not display any SCNs in the sections available for routine histology, and neither did we observe post-cytological findings adjacent to the parathyroid gland, such as fibrosis, hemorrhage, and/or calcifications—making the suspicion that the SCNs were artificially implanted in the parathyroid during fine-needle aspiration biopsy of the thyroid gland very unlikely.

## Discussion

SCNs of the thyroid gland are recurrently observed upon histological assessment and rarely constitute a diagnostic challenge due to the specific and recognizable phenotype of the lesions [2–10]. SCNs of the parathyroid gland, however, have to our knowledge never previously been reported across the scientific literature nor mentioned in classical works covering the parathyroid field, and hence could cause diagnostic difficulties given the assumed rarity of the phenomenon [18]. Indeed, the finding initially gave rise to an internal discussion within the endocrine pathology team at our unit whether or not the focal findings could constitute a metastatic deposit from squamous cell carcinoma (SCC) of the ear, throat, and neck region. However, the clear-cut immunohistochemical profile as well as the lack of histological SCC attributes (such as abundant mitoses) excluded this theory. More specifically, the weak GATA3 immunoreactivity observed argues in favor for SCNs, as this marker is positive in most of these lesions. [11]. Additional potential discriminators between SCNs and metastatic SCC, including a Ki-67 proliferation index, were unfortunately not retrievable in our case due to the cutting-away of the area of interest. The same was true for other markers such as galectin-3 and Bcl-2, which are both consistently positive in SCNs and could have been useful in supporting the diagnosis further [10]. Distant metastases to the parathyroid glands are rare and most often derive from adenocarcinoma primarily located in the breast, lung, or thyroid gland [19]. SCC distant metastases to the parathyroid glands have to our knowledge not yet been reported in the literature [19], and no case was reported among our own tumor database of > 4000 parathyroid tumors and normal parathyroid glands diagnosed at our institution between 1992 and 2017.

Theoretically, in our case, the close embryological association between the superior parathyroid glands and the UBB could imply that the two structures were in close embryonic contact during the migration towards the thyroid bed, alternatively that the UBB aberrantly implanted in the superior parathyroid when simultaneously seeding the thyroid parenchyma with cells. If the intra-parathyroidal SCNs in fact were functional or carried a physiological role in our case remains speculative, but the cells were negative for PTH using immunohistochemistry—which would argue against a hypothetical hormonal role.

We conclude that parathyroid SCNs exist, but probably as an exceedingly infrequent entity. Whether the finding represents a cell population with one or several physiological roles in the parathyroid gland or simply an ultimobranchial heterotopia aberrancy stemming from faulty migration during early development remains obscure—although the sheer rarity of the phenomenon would argue for the latter.
